# Melamine Sponge Functionalized with Urea-Formaldehyde Co-Oligomers as a Sorbent for the Solid-Phase Extraction of Hydrophobic Analytes

**DOI:** 10.3390/molecules23102595

**Published:** 2018-10-10

**Authors:** María Teresa García-Valverde, Theodoros Chatzimitakos, Rafael Lucena, Soledad Cárdenas, Constantine D. Stalikas

**Affiliations:** 1 Grupo FQM-215, Departamento de Química Analítica, Instituto Universitario de Investigación en Química Fina y Nanoquímica (IUIQFN), Universidad de Córdoba, Campus de Rabanales, Edificio Marie Curie, E-14071 Córdoba, Spain; q72gavam@uco.es (M.T.G.-V.); rafael.lucena@uco.es (R.L.); scardenas@uco.es (S.C.); 2 Laboratory of Analytical Chemistry, Department of Chemistry, University of Ioannina, 45110 Ioannina, Greece; chatzimitakos@outlook.com

**Keywords:** solid-phase extraction, melamine sponge, urea-formaldehyde co-oligomers, HPLC-DAD

## Abstract

A new procedure for the functionalization of melamine sponge (MeS) with urea-formaldehyde (UF) co-oligomers is put forward. The procedure differs from the typical synthesis of the UF co-polymer, as it employs a base-catalyzed condensation step at certain concentrations of urea and formaldehyde. The produced melamine-urea-formaldehyde (MUF) sponge cubes are hydrophobic, despite the presence of hydrophilic groups in the oligomers. The MUF sponge developed herein is used as a sorbent for the solid-phase extraction of 10 analytes, from 6 different classes (i.e., non-steroidal anti-inflammatory drugs, benzophenones, parabens, phenols, pesticides and musks) and an analytical method is developed for their liquid chromatographic separation and detection. Low limits of quantification (0.03 and 1.0 μg L^−1^), wide linear ranges and excellent recoveries (92–100%) are some of the benefits of the proposed procedure. The study of the synthesis conditions of MUF cubes reveals that by altering them the hydrophilic/lipophilic balance of the MUF cubes can be tuned, hinting towards a strong potential for many other applications.

## 1. Introduction

Sample pretreatment is an essential part of any analytical method, whatever technique is subsequently applied, as it has major effect on the quality of the results obtained. Sample pretreatment methods are constantly being developed to respond to the large variety of sample sources and compositions [1]. Solid-phase extraction (SPE) is a highly useful sample preparation-pretreatment technique. Many of the problems associated with liquid/liquid extraction, such as incomplete phase separations, less-than-quantitative recoveries and disposal of large quantities of organic solvents can be prevented with SPE. Moreover, SPE helps meet the sample preparation challenges, with respect to analyte pre-concentration and sample clean-up [2,3,4].

In recent years, interconnected microporous sorbent materials with favorable (super)hydrophobic surface properties and excellent sorption capacity, have attracted significant interest for potential applications. A water droplet on such a sorbent surface can roll off, even at inclinations of only a few degrees, while still retaining oil contaminants encountered on its way into its matrixes [5,6]. Motivated by these properties, many efforts have been devoted to developing novel and advanced 3D porous materials, which possess hydrophobic surface properties and interconnected macro-porous structures, with excellent sorption performance, for the separation and recovery of organic pollutants from water [7,8,9]. It may be mentioned that such efforts in developing functional architectural materials have significantly improved the sorption performance towards various organic solvents. Melamine sponge (MeS) is a three-dimensional, low-density, foam-like material made of a formaldehyde-melamine-sodium bisulfite copolymer. It has an open-hole structure, high porosity (>99%), excellent wettability and negligible cost to obtain [10,11] Because of the presence of functional groups, MeS is amenable to functionalization in order to tune its hydrophilicity or render it hydrophobic [12,13,14]. Such modifications have enabled the application of MeS-based hydrophobic materials to oil-absorption and oil/water separation [15,16,17].

Recently, we proposed for the first time, two modifications of MeS: first, with graphene in a one-step, fast procedure and we utilized it for the microextraction of sulfonamides from food and environmental matrixes [18] secondly, with copper sheets as a decorated material for the same purpose, capitalizing on the affinity of sulfonamides for copper, the rapid mass transport and on the low back-pressure due to their macropore and throughpore structure [19].

Melamine-urea-formaldehyde (MUF) resins are used in the manufacturing of water-resistant particleboards, papers and laminates. The chemistry of melamine reacting with formaldehyde and urea-formaldehyde (UF) is well known [20]. A molecularly imprinted monolithic resin has, also, been synthesized from MUF via an in-situ polymerization [21], which showed good water compatibility and excellent molecular recognition of plant growth regulators, when it was applied as an SPE sorbent. Melamine-urea-formaldehyde introduces abundant hydrophilic groups (such as hydroxyl, carboxyl, imino and amino groups) into the polymer and improves its compatibility with aqueous solutions and rapid mass transport.

The purpose of the study herein is to develop a novel, three-dimensional, low-density sponge-based hydrophobic material by properly developing formaldehyde-urea co-oligomers on the surface of a melamine sponge. This is achieved by carefully tuning the hydrophilic/lipophilic balance in favor of the latter. Melamine provides the backbone for the development of the oligomers. The modified melamine sponges are then used as an SPE material for the extraction of hydrophobic molecules belonging to six different classes of analytes.

## 2. Results and Discussion

### 2.1. Synthesis Optimization

The synthesis consisted of heating a urea-formaldehyde solution at alkaline pH for the formation of UF oligomers (*vide infra*) and their attachment to melamine to form the modified 3D material. Typically, UF polymer synthesis has been reported to consist of two steps: first, polymerization under alkaline conditions and secondly, condensation under acidic conditions [22]. However, this procedure leads to a highly hydrophilic polymer, owing to the occurrence of many hydrophilic groups (such as hydroxyl, carboxyl, imino and amino groups). In order to obtain a more hydrophobic product, in the present study, we employed a base-catalyzed UF condensation step, during which the formation of the products was kinetically and thermodynamically controlled. Under these conditions, the typical resin structure was not generated, while oligomers were primarily formed [23]. The MUF sponge synthesis was optimized so as to achieve maximum adsorption of the analytes. The ratio of formaldehyde: urea and the concentrations of both reagents were optimized with the purpose of ensuring efficient functionalization of MeS. Moreover, the number of sponges that can be produced in a single functionalization batch was studied. In all cases, the criterion used for the evaluation of the produced MUF cubes was the total extraction yield from an aqueous solution containing a mixture of analytes (50 μg L^−1^ each). A complete list of the analytes, their structure and some physicochemical characteristics are given in Table 1.

#### 2.1.1. Effect of Formaldehyde-to-Urea Ratio

In order to study the formaldehyde-to-urea ratio (F/U), the amount of urea in the mixture was held constant at 0.88 mmol and formaldehyde was varied to probe its effect on the resulting MUF cubes. To this end, different products were obtained using F/U molar ratios of 1.0, 1.4, 1.6 and 2.0. Extraction of the analytes was carried out as explained in Section 3.4. According to the results (data not shown), the MUF cubes prepared at molar ratios of 1.0, 1.4 and 1.6 had similar extraction behavior for all analytes (the variations were lower than 7%). When a molar ratio of 2.0 was used, the total extraction yield was decreased by nearly 10%. This can be attributed to the prevalence of ether linkages (–CH_2_–O–CH_2_–) over methylene (–CH_2_–) ones, at this ratio [23], which was unsuitable for producing MUF cubes with reasonable extraction yields for the target compounds. Ether bridges endow oligomers with hydrophilicity, compared to methyl analogues, which are undesirable in our case, since target compounds are adsorbed mainly via hydrophobic interactions (*vide infra*). Among the three other molar ratios with similar extraction behavior, that of 1.0 was finally opted for, because reproducible formation of a linear chain UF oligomer was favored in contrast to the branched UF oligomer structures produced at higher ratios [22,23].

#### 2.1.2. Effect of Concentrations of Urea and Formaldehyde

To maximize the extraction potential of the MUF cubes, the concentrations of urea and formaldehyde were studied in depth. Different MUF cubes were synthesized using 0.44, 0.88, 1.76 and 3.52 mmol of each of the reagents. The results are summarized in Table S1 (Supplementary Materials). It was observed that as the amount of the reagents increased, the extraction yield slightly decreased for all analytes (from 0.44 to 3.52 mmol the adsorption decreased by nearly 10%). Moreover, it was observed that the hydrophobicity of the MUF cubes slightly decreased as the amount of reagents increased. To verify this finding, MUF cubes were prepared using 13.2 mmol of each reagent (30 times higher than the lower tested amount). It was found that these cubes were highly hydrophilic and their sorptive behavior was quite different from that of the proposed MUF cubes. Specifically, the hydrophilic MUF cubes were able to adsorb low percentages of FEN, FLU, BPB and BP8 (<20%) while the rest of the analytes were not adsorbed at all. This is probably due to the ionizable nature of the adsorbed compounds, which can interact with the carboxyl and amino groups of the UF oligomers, albeit to a lesser extent than with MUF cubes prepared using the proposed low reagent concentrations. High concentrations of the reagents favor the formation of branched structures, that contain fewer free surface groups and result in lower extraction yields [22,23]. This, in turn, bespeaks that all analytes are adsorbed primarily via hydrophobic interactions.

As mentioned above, the products of the base-catalyzed UF condensation step are kinetically and thermodynamically controlled. The formation of methylene ether bridges (a kinetically favorable process) is preferred to that of methylene bridges (a thermodynamically favorable process) [22,23]. This signifies that a hydrophilic/lipophilic balance of the oligomers can be maintained, which affects directly the prepared MUF cubes and depends on the quantities and ratios of the reagents used. When the amount of each reagent was lower than 0.44 mmol, the extraction yield of the MUF cubes was decreased, probably, as a result of the lower amount of UF oligomers attached to the MeS. As an optimum, the amount of each reagent was selected to be 0.44 mmol, satisfying both the best performance and the minimum reagent amount.

#### 2.1.3. Number of Synthesized Sponge Cubes per Batch

Until now, single MUFs were prepared to examine the aforementioned conditions. However, the production of more than one cube per batch is a great advantage in terms of time and cost. For this reason, five MUF cubes were synthesized, simultaneously, in the same batch, by increasing proportionally the amounts of the reagents in the reaction mixture (i.e., from 0.44 mmol in 20 mL water per sponge to a total of 2.2 mmol in 100 mL of water). The extraction performance of the MUF cubes originating from a big batch was compared with that from the single-batch MUF cubes. The results revealed that no differences were recorded when bulk synthesis was carried out with more than one sponge at a time. Additionally, we examined the reproducibility of the extraction yield using MUF cubes from big batch syntheses, conducted on different days. In this case, also, the differences between various (big) batches from three consecutive days were insignificant (<6%). Therefore, five cubes were selected to be simultaneously modified according to the procedure.

### 2.2. Characterization of MUF Sponges

Figure S1 (Supplementary Materials) shows the SEM micrographs of raw MeS and MUF sponges. It can be seen that after functionalization, the fibrous 3D structure is maintained. The fact that bulk structures of UF are not observed strengthens the notion that using the base-catalyzed condensation step, oligomers are formed. Moreover, the typical UF resin structure was not observed. The porous framework of the cubes seems to shrink compared to the bare melamine analogues. This is verified by the reduction in cube size to nearly 0.9 × 0.9 × 0.9 cm (from 1 × 1 × 1 cm).

**Figure 1:**
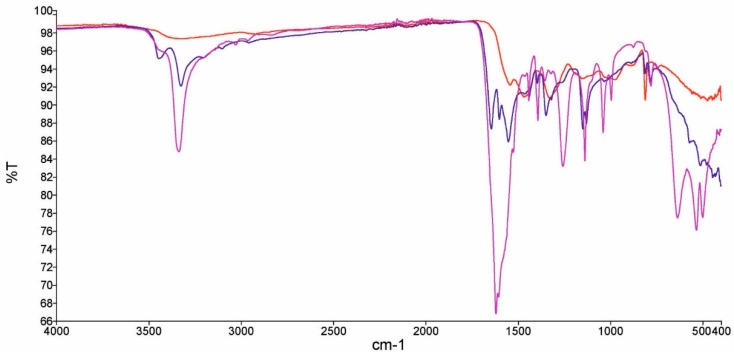
FTIR spectra of melamine sponge (MeS) (red line), urea-formaldehyde (UF) polymer (purple line) and melamine-urea-formaldehyde (MUF) cubes (blue line).

The FTIR spectra of pristine MeS, MUF cubes and UF resin can be seen in Figure 1. It is evident that after functionalization the IR spectrum of MUF cubes has some marked differences compared to that of MeS, evidencing the functionalization of MeS with UF, which differentiate it from typical UF resin. In contrast, the similarities suggest the successful functionalization of MeS with UF. The peaks at 1000, 1050 and 1250 cm^−1^ (ether C–O stretch) do not appear in the spectrum of MUF, signifying that ether groups are not present, while the peak around 1450 cm^−1^ is due to –CH_2_ bending. The spectrum of UF polymer shows a large peak around 1650 cm^−1^, due to amide group absorption. In the case of MeS and MUF cubes, the absorption bands around the above area are much weaker compared to those of UF polymer, suggesting that the amide groups in MUF cubes are limited. This supports the hydrophobic character of MUF cubes and strengthens our assertion that hydrophobic oligomers are formed under the stated synthesis conditions.

**Figure 2:**
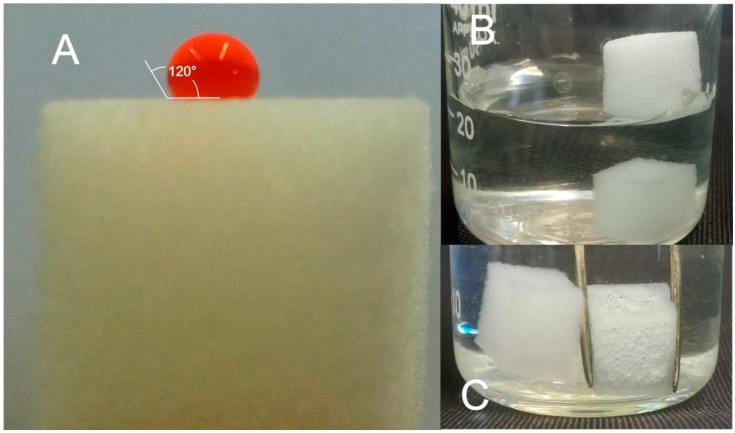
(**A**) A red colored water drop on the surface of a MUF cube and the approximate contact angle, (**B**) a MUF cube (upper) and a MeS (lower) in a glass beaker filled with water and (**C**) a MUF cube (right) immersed in water using forceps next to bare MeS (left).

The wettability of the material was evaluated with the contact angle, which is a parameter that indicates the degree of wetting when a solid and liquid interact. Figure 2A shows a MUF cube, for which a red-colored (for visualization purposes) water drop was placed on the surface. The drop on the MUF sponge had an almost spherical shape with a contact angle of around 120°, demonstrating the hydrophobicity of the material. Moreover, when a MUF cube was placed in a glass beaker filled with water, the cube floated on the surface of the water, in contrast to bare MeS, which submerged easily to the bottom (Figure 2B). When MUF cubes were immersed in the water using metal forceps, the surface of the cube was like a silver mirror, consisting of many air bubbles, which is also characteristic of the hydrophobicity of the material (Figure 2C) [18,19].

### 2.3. Optimization of the Proposed Procedure

#### 2.3.1. Effect of Sample pH and Ionic Strength on Extraction Yield

**Figure 3:**
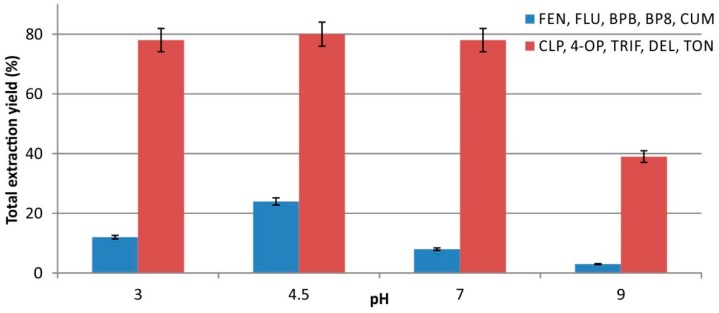
Effect of sample pH on the total extraction yield of the examined analytes, using the MUF cubes.

Due to the variety of classes of analytes employed in this study, a screening experiment in a broad pH range (i.e., 2.0–9.5) was carried out in order to elucidate the effect of pH on the extraction performance. The performance was improved at pH 4.5 for FEN, FLU, BPB, BP8, and CUM, while at pH 9.5 these analytes were hardly adsorbed (<5%). This is due to the ionizable nature of these compounds, hinting that their adsorption is not because of purely hydrophobic interactions. With regard to the rest of analytes, the variations in their extraction throughout the pH range of 2.0–7.0 where negligible, while a pronounced decrease by nearly 40% was noticed at pH 9.5. Figure 3 depicts the total extraction yield at different pH values, showing a favorable behavior at pH 4.5. As mentioned above, the adsorption of the analytes was achieved mainly via hydrophobic interactions and to a lesser extent (for some analytes) via other kind of interactions. For the subsequent experiments, the pH of the aqueous samples was adjusted to 4.5.

The effect of ionic strength on the extraction performance was evaluated using sodium chloride as model electrolyte. The concentrations tested were 0.25%, 0.5% and 1.0% *w*/*v*. The results showed that as the concentration of sodium chloride increases the extraction yield decreases. The addition of 0.25% and 0.5% *w*/*v* of salt reduced the total extraction yield from 29% to 18.5%, while 1.0% *w*/*v* of salt, further reduced adsorption to 13%. Although, commonly, salting out enhances the extraction yield of hydrophobic compounds, this was not the case in our method. This may be either due to the reduction of the diffusion rate of analytes, which, in general, is more pronounced for less polar analytes, or to the fact that increased salt concentrations cause an increase to the strong degree of sorption of the hydrophobic components, hindering their extraction [24,25]. Therefore, the deliberate increase of ionic strength of a sample prior to analysis is not beneficial.

#### 2.3.2. Effect of Adsorbent Quantity and Sample Volume

The sorbent amount was studied by increasing the number of standard MUF cubes placed in the SPE cartridge, under constant flow rate (ca. 0.6 mL min^−1^) with a sample volume of 25 mL. For this reason, one, two and three sponges were used simultaneously for the extraction of the analytes. At this step, elution was conducted by increasing proportionally the amount of the solvent used (500 μL methanol per cube), so that the observed differences were attributed solely to the extraction step. The performance of the extraction was improved as the number of sponges increased. Almost 15% and 25% increases were achieved with the second and the third MUF cubes, respectively, due to the marked increase in the available surface and sorbent functional groups of MUF. Adding a fourth MUF cube in the extraction unit was not found to further increase the extraction yield of the proposed procedure. Thus, three cubes were thereafter employed in order to achieve optimum extraction.

Regarding sample volume, experiments were carried out with different volumes spiked either with the same concentration of analytes, or with the same constant quantity of them. In both cases, the tested sample volumes were 10, 25, 50 and 75 mL. In the case of constant spiked quantity, the results showed a trivial decrease in the extraction efficiency of the target analytes (<7%) when sample volume was increased from 10 to 50 mL. In contrast, the extraction efficiency was lower by ~15% when the sample volume was 75 mL. In the case of different sample volumes, by keeping constant the concentration of analytes, the extraction efficiency was the same for 10 and 25 mL. A decrease of approximately 20% and 35% was noticed when the sample volume rose from 50 to 75 mL (see Figure S2 of Supplementary Materials). According to the above results, a sample volume of 25 mL was selected as the optimum, so as to attain significant enrichment factors of the analytes, with the proposed procedure.

#### 2.3.3. Elution Conditions

Methanol and acetonitrile were tested as eluents for the proposed sample preparation method and the analytes of concern. Using methanol, 40% of the extracted analytes was eluted while acetonitrile was able to elute approximately 30%. As the extraction was found to be pH-dependent, we examined the presence of formic acid or ammonia in the eluent (in both cases 2% *v*/*v* was added). When formic acid was added no significant differences were noticed for any analyte. However, when ammonia was added, elution yield sharply decreased by nearly 20% for FEN and FLU; for the rest of analytes, a trivial reduction of ~5–8% was noticed. Therefore, neither formic acid nor ammonia was finally added to methanol, which was selected as the optimum organic solvent.

As only 40% of the analytes were extracted under the above conditions, we examined the possibility of increasing this percentage by studying the elution volume. Preliminary experiments showed that an elution volume of less than 1 mL was not efficient when three sponges were used for the extraction. Therefore, 1 and 2 mL of methanol were subsequently examined. The elution yield was almost double when 2 mL of methanol were used (it was overall ~80%). Still, the analytes were not eluted completely from the sponges. Therefore, we conducted a second elution step with additional 2 mL of methanol following the first elution and we found that the analytes were totally recovered. In order to minimize the elution volume, the 2 mL of methanol already used for the first elution was passed, again, through the column and it was found that the extraction yield increased by 10%. A third elution step was conducted with the same eluent and the analytes were found to be removed completely from the cubes. Instead of using a total of 4 mL of methanol, we ended up using only 2 mL, reducing the solvent and the amount of time needed to evaporate the eluent using a nitrogen stream (from ~50 to ~35 min).

### 2.4. Method Validation

Under the aforementioned optimum conditions, the analytical figures of merit of the proposed procedure were studied. The analytical characteristics can be seen in detail, in Table 2. Calibration plots were drawn for each analyte, with the lowest concentrations as the limits of quantification (*LOQ*). Linear responses were noticed for all analytes up to a concentration of 100 μg L^−1^. The coefficients of determination (*R*^2^), in all cases, were higher than 0.9990, bespeaking satisfactory linearities. The limits of detection were between 0.01 and 0.33 μg L^−1^. Enrichment factors (*EF*) and extraction percentages (*E%*) were calculated using the following equations:

(1) $$\;EF=\frac{C_{el}}{C_{aq}}\;$$

(2) $$\;E\%=\frac{C_{el}}{C_{el}+C_{aq}}\;$$

where, *C_el_* and *C_aq_* are the analyte concentrations in the eluent and the aqueous sample, respectively [26]. The values were found to be between 22% and 30% and above 96%, respectively, for all tested compounds. The precision of the method was calculated as the within-day and between-day relative standard deviation (*RSD*) by analyzing five different samples within the same day and three different samples on five consecutive days, respectively. The samples were spiked with the lowest concentrations employed for drawing the calibration plots. Within-day and between-day *RSD* were found to be between 5.6–7.3% and 6.1–8.4%, respectively.

### 2.5. Analysis of Real Samples

In order to examine the applicability of the proposed procedure, relative recoveries were calculated by analyzing lake water samples spiked with the analytes at two concentration levels, i.e., 3 and 10 times the *LOQ*. Typical chromatograms of a blank and a spiked lake water sample with a mixture of analytes (50 μg L^−1^ each) are given in Figure S3 of the Supplementary Materials. The recoveries (summarized in Table 2) were in the ranges of 92–98% and 96–100% for the low and high tested concentrations, respectively. These values, along with *RSDs* and *LOQs* are comparable with those reported by other works, signifying that the proposed procedure is an excellent alternative method. A complete comparison with other methods can be seen in Table 3.

## 3. Materials and Methods

### 3.1. Chemical and Reagents

The reagents were at least of analytical grade and were purchased from Sigma-Aldrich (Steinheim, Germany). Stock standard solutions of the analytes, fenbufen (FEN), butylparaben (BPB), benzophenone-8 (BP8), 4-cumylphenol (CUM), flurbiprofen (FLU), chlorpyrifos (CLP), trifluralin (TRIF), 4-octylphenol (4-OP), tonalide (TON), and deltamethrin (DEL) were prepared in methanol at a concentration ranging from 3 to 20 g L^−1^ and were stored at −18 °C. Working solutions were prepared daily in double distilled water (DDW). Melamine sponges were bought from a local store in Ioannina (Greece).

### 3.2. Synthesis of Urea-Formaldehyde Sponges

Melamine sponges were firstly cut into cubes of 1 cm^3^ (1 × 1 × 1 cm), rinsed with ethanol and left to dry. The cubes were coated with urea-formaldehyde (UF) oligomers in a simple procedure. In brief, 0.132 g of urea (2.2 mmol) dissolved in 100 mL of DDW was mixed with 66.1 µL of formaldehyde (2.2 mmol) and the pH value was adjusted to 10 with NaOH 1 M. Then, five sponge cubes of ca. 10 mg each were added to the solution and stirred at 85 °C, for 2.5 h. The temperature was decreased to 45 °C and 2.0 mL of methanol was added dropwise to cease the polymerization process and the solution was kept under stirring for a further 10 min. Finally, the solution was cooled to room temperature and the MUF cubes were removed, squeezed to remove excess solution and washed with DDW and ethanol. After the washing step, cubes were dried at 60 °C for 8 h.

### 3.3. Apparatus

The morphology of MUF cubes was observed by scanning electron microscopy (SEM). Images were obtained using a JEOL JSM 6300 microscope at the Central Service for Research Support of the University of Córdoba. Chromatographic analysis was carried out on a Shimadzu (Kyoto, Japan) HPLC system coupled to a SPD-M20A Diode Array Detector (DAD). The column used for the separation was a Hypersil ODS (250 × 4.6 mm, 5 µm particle size) from Thermo Fisher Scientific (Waltham, MA, USA), kept at 25 °C in a CTO 10AS column oven. Samples were injected using a Rheodyne injector with a sample loop of 20 µL volume. The mobile phase consisted of water (A) and acetonitrile (B), containing 0.1% (*v*/*v*) formic acid. The analytes were separated following a gradient elution program from 55% to 80% B in 30 min and then to 85% in 5 min. The total chromatogram time was 35 min. Mobile phase was delivered using a LC20AD pump, at a flow rate of 0.8 mL min^−1^. The detector was set at a wavelength range of 200–360 nm. Data acquisition and processing were carried out using a LC-solution software version 1.21. FTIR spectra were recorded on a Spectrum Two FTIR using an attenuated total reflectance accessory (PerkinElmer, Cambridge, MA, USA). Approximate contact angle measurements were carried out by analyzing various photographs of water droplets on MUF cubes using the Corel Draw X6 software.

### 3.4. SPE Procedure

For the extraction of the analytes, three MUF cubes of the dimensions mentioned above were placed in a SPE cartridge (inner diameter: 0.8 cm), which was attached to a flow control valve (Luer stopcock). The extraction unit accommodated the need for the extraction and final elution step of the analytes and allowed the interaction of sorbent with the sample, at a controlled flow rate of 0.6 mL min^−1^. Before extraction, the cubes were preconditioned with 3 mL of methanol followed by 6 mL of DDW acidified to pH 4.5 with hydrochloric acid. A sample aliquot of 25 mL, the pH of which was previously adjusted to 4.5, was passed through the extraction unit, at a rate of ca. 0.5 mL min^−1^. Then, 5 mL of DDW water (pH 4.5) was passed through the cubes, under the same flow rate as in the cleaning step and the cubes were dried using a nitrogen stream. Finally, the analytes were eluted using 2.0 mL of methanol, which was collected and passed through the sponges three times, evaporated up to 100 μL, under a gentle nitrogen stream and was injected into the HPLC-DAD system.

## 4. Conclusions

In this study, a novel functionalization procedure of MeS is applied. Adopting a base-catalyzed condensation step, which is fundamentally different from that for the typical UF resin synthesis, we obtained UF co-oligomers, that were developed on the surface of MeS. The resulting MUF cubes were highly hydrophobic, owing to the procedure by which the UF oligomers were formed. By altering the parameters that affect the synthesis, the hydrophilic/lipophilic balance of the functionalized cubes can be tuned. Furthermore, capitalizing on the hydrophobic properties of the synthesized MUF cubes we developed an SPE procedure, suitable for hydrophobic analytes. The analytical method achieves low LOQs, satisfactory recoveries, good reproducibility and wide linear ranges. Overall, the developed material can serve as an excellent alternative sorbent for classical SPE procedures.

## Figures and Tables

**Table 1:** Structure and physicochemical properties of the studied analytes.

Compound	Category	Structure	Log *P*	p*K*_a_	Quantification Wavelength	Retention Time (min)
Fenbufen (FEN)	Non-steroidal anti-inflammatory drugs	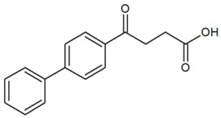	3.13	4.22	285	7.2
Flurbiprofen (FLU)		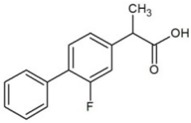	4.11	4.42	254	17.2
Benzophenone-8 (BP8)	Benzophenones	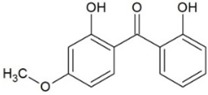	3.93	7.11	285	12.0
Butylparaben (BPB)	Parabens	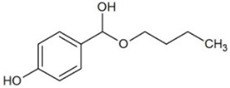	3.46	8.47	254	9.8
Cumylphenol (CUM)	Phenols	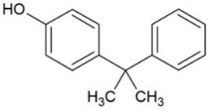	4.17	10.0	275	15.3
4-octylphenol (4-OP)		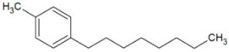	5.66	10.15	275	24.8
Chlorpyrifos (CLP)	Pesticides	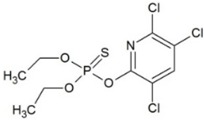	4.77	-	285	18.4
Trifluralin (TRIF)		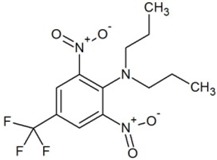	5.41	-	285	20.6
Deltamethrin (DEL)		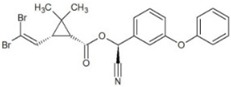	6.20	10.62	275	31.1
Tonalide (TON)	Musks	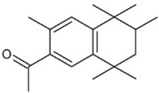	5.70	-	254	27.2

**Table 2:** Analytical figures of merit of the developed solid-phase extraction procedure, using MUF cubes ^a^.

Compound	Linear Equation	Coefficient of Determination (*R*^2^)	*LOD* (μg L^−1^)	*EF*	*E%*	*RSD* (%)		Relative Recoveries (%)	
						Within-Day (n = 5)	Between-Day (n = 3 × 5)	3 × *LOQ*	10 × *LOQ*
Fenbufen	y = 31,481x + 1117	0.9998	0.02	22	96	7.0	7.4	94	96
Butylparaben	y = 30,462x + 642	0.9993	0.01	23	96	6.7	7.4	95	98
Benzophenone-8	y = 30,121x + 832	0.9992	0.02	27	96	7.1	7.8	96	99
Cumylphenol	y = 30,432x + 667	0.9991	0.02	23	96	6.4	7.1	95	98
Flurbiprofen	y = 29,010x + 1844	0.9995	0.02	25	96	6.7	8.4	93	97
Chlorpyrifos	y = 7498x + 1091	0.9993	0.09	24	96	5.6	6.1	94	96
Trifluralin	y = 30,967x + 967	0.9996	0.02	25	96	6.9	8.0	97	99
4-octylphenol	y = 30,367x + 784	0.9997	0.02	24	96	6.4	7.3	98	100
Tonalide	y = 29,633x + 518	0.9998	0.01	23	96	7.3	8.2	92	98
Deltamethrin	y = 2321x + 418	0.9994	0.33	30	97	5.7	7.1	96	98

^a^ Abbreviations: *LOD*: limit of detection, *EF*: Enrichment factor, *E%*: extraction efficiency, *RSD*: relative standard deviation.

**Table 3:** Comparison of the performance of the developed procedure with methods reported in literature ^a, b^.

Analytes	Matrix	Method	Material	Sample Volume (mL)	Elution Volume (mL)	Equipment	*RR* (%)	*RSD* (%)	Ref.
BP8	Swimming pool water	SPE	C18	100	3	GC-MS	-	<3% (*CV*)	[27]
BPB	River water	SPE	Oasis HLB	500	4	HPLC-MS/MS	92.9	15.5	[28]
FEN and FLU	Plasma and urine	MSPE	G/Fe_3_O_4_	5	0.5	HPLC-PDA	97.5–102	2–4.2	[29]
CUM and 4-OP	Water	SPE	Oasis HLB	500	6	HPLC-MS/MS	-	-	[30]
CLP, DEL and TRIF	Wetland sediments	SPE	Oasis HLB	500	5	GC-ECD	69–101	<15	[31]
FEN, BPB, BP8, CUM, FLU, CLP, TRIF, 4-OP, TON and DEL	Lake water	SPE	MUF	25	5	HPLC-PDA	92–100	5.6–8.4	This work

^a^ Methods for the determination of the analytes using sample processing and chromatographic separation are compared. ^b^ Abbreviations: *RR*, relative recovery; *RSD*, relative standard deviation; *CV*, coefficient of variation; FEN, fenbufen; BPB, butylparaben; BP8, benzophenone-8; CUM, cumylphenol; FLU, flurbiprofen; CLP, chlorpyrifos; TRIF, trifluralin; 4-OP, 4-octylphenol; TON, tonalide; DEL, deltamethrin; SPE, solid-phase extraction; MSPE, magnetic solid-phase extraction; MUF, melamine urea formaldehyde.

## Supplementary Materials

The following are available online. Table S1: Total amounts of urea and formaldehyde used for the synthesis of MUF cubes and the respective total extraction yields of the examined analytes, Figure S1: SEM micrographs of (A) MeS and (B) MUF cubes, Figure S2: Effect of various sample volumes (spiked with constant amount or the same concentration of analytes) on the extraction efficiency of the method, Figure S3: Chromatograms (at 285 nm) of a blank lake water sample (lower chromatogram) and a lake water sample spiked with 50 μg L^−1^ of the analytes (upper chromatogram). Abbreviations: FEN, fenbufen; BPB, butylparaben; BP8, benzophenone-8; CUM, cumylphenol; FLU, flurbiprofen; CLP, chlorpyrifos; TRIF, trifluralin; 4-OP, 4-octylphenol; TON, tonalide; DEL, deltamethrin.
