# Supplementary material for: Melamine Sponge Functionalized with Urea-Formaldehyde Co-Oligomers as a Sorbent for the Solid-Phase Extraction of Hydrophobic Analytes
Source: Molecules. 2018 Oct 10;23(10):2595. doi: 10.3390/molecules23102595 (PMC6222532; doi:10.3390/molecules23102595)
Supplement: Supplementary file 1 [file molecules-23-02595-s001.pdf]

Supplementary Material
Melamine Sponge Functionalized with Urea-Formaldehyde Co-Oligomers as a Sorbent for the Solid-Phase Extraction of Hydrophobic Analytes

María Teresa García-Valverde [1], Theodoros Chatzimitakos [2], Rafael Lucena [1], Soledad Cárdenas [1], Constantine D. Stalikas [2,*]

[1] Grupo FQM-215, Departamento de Química Analítica, Instituto Universitario de Investigación en Química Fina y Nanoquímica (IUIQFN), Universidad de Córdoba, Campus de Rabanales, Edificio Marie Curie, E-14071 Córdoba, Spain; q72gavam@uco.es (M.T.G.-V.); rafael.lucena@uco.es (R.L.); scardenas@uco.es (S.C.)

[2] Laboratory of Analytical Chemistry, Department of Chemistry, University of Ioannina, 45110 Ioannina, Greece; chatzimitakos@outlook.com (T.C.)

[*] Correspondence: cstalika@cc.uoi.gr; Tel.: +302651008414

Table S1. Total amount of urea and formaldehyde used for the synthesis of MUF cubes and the respective total extraction yields of the examined analytes.

Total amount of reagents (mmol)	Total extraction yield (%)
0.44	38.4
0.88	36.4
1.76	33.6
3.52	29.2
13.2	3.3

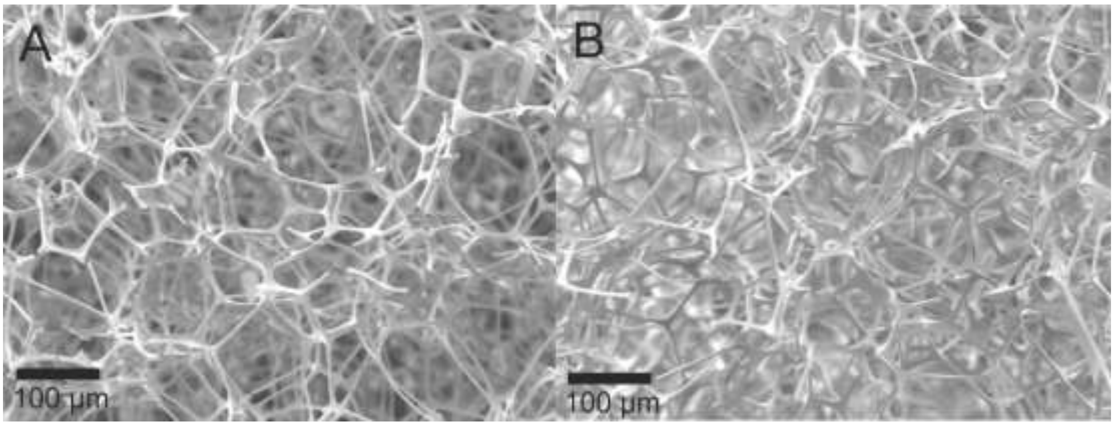

Figure S1. SEM micrographs of (**A**) MeS and (**B**) MUF cubes.

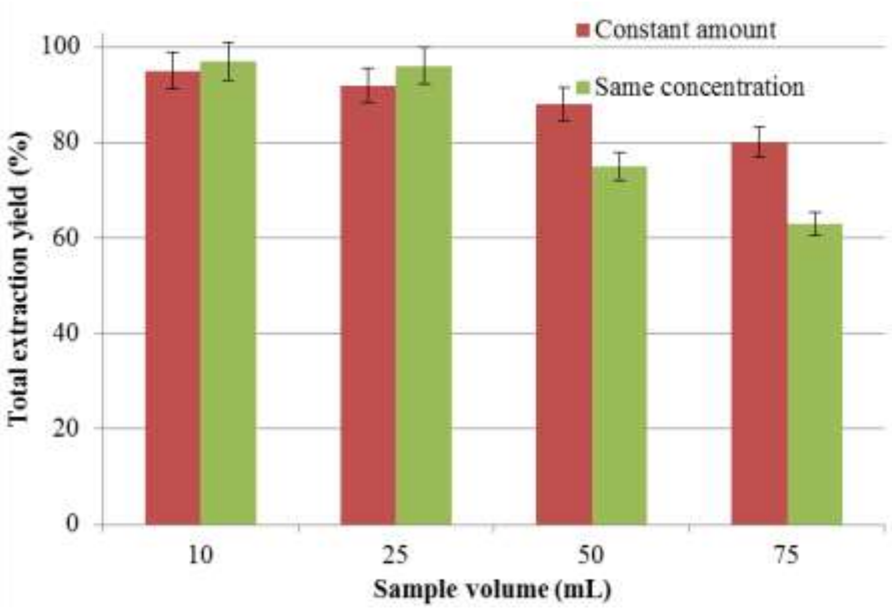

Figure S2. Effect of various sample volumes (spiked with constant amount or the same concentration of analytes) on the extraction efficiency of the method.

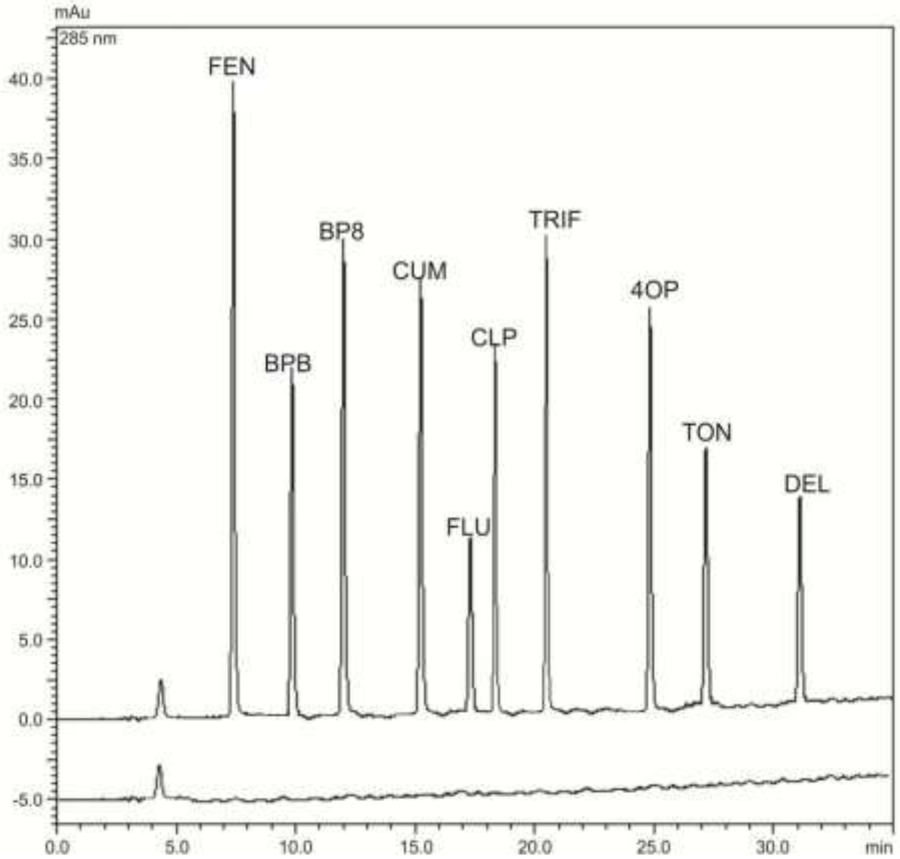

Figure S3. Chromatograms (at 285 nm) of a blank lake water sample (lower chromatogram) and a lake water sample spiked with 50 μg L^{-1} of the analytes (upper chromatogram). Abbreviations: FEN, fenbufen; BPB, butylparaben; BP8, benzophenone-8; CUM, cumylphenol;

FLU, flurbiprofen; CLP, chlorpyrifos; TRIF, trifluralin; 4-OP, 4-octylphenol; TON, tonalide; DEL, deltamethrin.
